# Operationalizing independent oversight in large-scale public health cohorts: Monitoring protocol for the NIMHANS–NH–SKAN Stroke project

**DOI:** 10.1371/journal.pone.0361702

**Published:** 2026-11-04

**Authors:** Mutharaju Arelingaiah, Arpitha Arun, Bangalore Sivilan Madhuri, Kibballi Madhukeshwar Akshaya, Huluvadi Shivalingaiah Anwith, Shivappa Usha, Ramesh Holla, Aravind Karinagannanavar, Malatesh Undi, Sharankumar Holyachi, Pradeep S. Banandur, Gautham Melur Sukumar, Banavaram Anniappan Arvind, Betagalli Mallappa Shivaraj, Khandiga Sharma Lakshmi, Thimappa Hegde, Komal Prasad, Lavanya Garady, Ankesh Ashwini, Upashana Medhi

**Affiliations:** 1 Department of Psychiatric Social Work, NIMHANS, Bengaluru, Karnataka, India; 2 Senior Research Officer, NIMHANS-NH-SKAN Stroke Project, NIMHANS, Bengaluru, Karnataka, India; 3 Senior Community Mobiliser, NIMHANS-NH-SKAN Stroke Project, NIMHANS, Bengaluru, Karnataka, India; 4 Department of Community Medicine, Yenepoya Medical College, Yenepoya (Deemed to be University), Mangalore, Karnataka, India; 5 Department of Community Medicine, Kempegowda Institute of Medical Sciences, Bengaluru, Karnataka, India; 6 Department of Community Medicine, Rajarajeswari Medical College and Hospital, Bengaluru, Karnataka, India; 7 Department of Community Medicine, Kasturba Medical College Mangalore, Karnataka, Manipal Academic of Higher Education, Manipal, Karnataka, India; 8 Department of Community Medicine, K. H. Patil Institute of Medical Sciences, Gadag, Karnataka, India; 9 Department of Community Medicine, Karwar Institute of Medical Sciences, Karwar, Karnataka, India; 10 Department of Community Medicine, Koppal Institute of Medical Sciences, Koppal, Karnataka, India; 11 Department of Epidemiology, NIMHANS, Bengaluru, Karnataka, India; 12 Department of Community Medicine, Chikkamagaluru Institute of Medical Sciences, Chikkamagaluru, Karnataka, India; 13 Department of Pathology, Sanjay Gandhi Institute of Trauma and Orthopaedics, Bengaluru, Karnataka, India; 14 Mazumdar Shaw Medical Centre, Narayana Institute of Neurosciences, Bengaluru, Karnataka, India; 15 Neurosurgeon, Narayana Institute of Neurosciences, Bengaluru, Karnataka, India; 16 SKAN Research Trust, Bengaluru, Karnataka, India; 17 Skan Project, NIMHANS, Bengaluru, Karnataka, India; 18 Senior Research Officer, Skan Project, NIMHANS, Bengaluru, Karnataka, India; Amrita Vishwa Vidyapeetham - Amritapuri Campus, INDIA

## Abstract

**Background:**

onitoring the implementation of observational studies are rare, unlike experimental studies. This article aims to describe the development and implementation of a monitoring plan and framework for a large-scale cohort and quasi-experimental study: the NIMHANS-NH-SKAN Stroke project.

**Methods:**

The monitoring framework was developed through a systematic, stepwise process involving expert consultation workshops conducted by an Independent Monitoring Team in collaboration with the study team. A logical framework approach was used to identify key processes throughout the study implementation. These processes were then disaggregated into critical activities (sub-processes) to generate an initial list of monitoring items. These items were prioritized using the Vital–Essential–Desirable (VED) strategy through consensus building. The shortlisted items were further refined using SMART criteria (Specific, Measurable, Achievable, Relevant, and Time-bound) to finalize checklist components. Key Performance Indicators were subsequently derived from the finalized checklist items. The framework emphasized usability, feasibility, and relevance to implementation, enabling systematic monitoring of critical study processes.

**Results:**

A total of 89 activities were identified and consolidated into 10 checklists, from which 16 Key Performance Indicators (KPIs) were derived. The monitoring framework primarily emphasized indicator-based assessment of project outputs and outcomes while ensuring adherence to key processes and quality standards. KPIs were expressed as proportions to facilitate standardized performance assessment across sites and time periods. The checklists comprised a combination of in-person observations and record reviews. Monitoring findings were synthesized into periodic reports based on KPI proportions, enabling objective evaluation of implementation performance and identification of areas requiring corrective action.

**Conclusions:**

Timely and systematic monitoring is critical to ensure rigor in large-scale projects, and the approach described in this study provides a replicable framework for the development of robust monitoring systems in similar community-based large-scale observational studies in India and similar countries.

## Introduction

Large-scale public health surveys and cohort studies generate essential, policy-relevant data that inform health planning, service delivery, and research priorities [1,2]. Despite their value, the complexity and scale of such studies make them susceptible to operational, methodological, and ethical challenges. These may result in protocol deviations, inconsistent field implementation, and compromised data quality [3]. To address these concerns, structured and independent monitoring systems have emerged as a key strategy for enhancing the validity and reliability of studies [4].

Internationally, monitoring frameworks have evolved since the 1960s to ensure ethical conduct, participant safety, and rigorous data management in large research platforms [5,6]. Currently, many long-term observational cohorts and biobank studies are monitored for implementation and compliance, in accordance with guidance from independent ethics and governance councils [7]. As a result, structured monitoring has become an integral component of both academic and industry-sponsored research, enabling unbiased oversight and supporting the generation of high-quality evidence [8–11].

In India, large-scale population health initiatives, such as the National Family Health Survey (NFHS), the National TB Elimination Program (NTEP), and the National AIDS Control Program (NACP), employ well-defined internal and external monitoring mechanisms to ensure uniformity and data accuracy across diverse settings [4,12,13]. However, these systems are primarily oriented towards field verification, back-checks, supervisory review, and laboratory quality assurance [14–16]. They do not typically incorporate independent, multi-domain oversight comparable to global monitoring frameworks. Existing national and international monitoring frameworks have established robust approaches for ensuring data quality, program performance, and protocol compliance within their intended settings. However, these frameworks are largely designed for cross-sectional surveys, disease-specific programs, or clinical trials and do not comprehensively address the monitoring requirements of implementation studies involving community mobilization, longitudinal cohort follow-up, laboratory procedures, hospital preparedness, study fidelity, and data management. These components must be assessed simultaneously to safeguard data integrity and participant protection [17]. These gaps underscore the need to develop a comprehensive implementation monitoring framework tailored to the study, given its multiple operational domains.

The NIMHANS–NH–SKAN (National Institute of Mental Health and Neuro Sciences–Narayana Hrudayalaya–SKAN Research Trust) stroke study aims to investigate stroke-related risk factors, incidence, management pathways, and outcomes with the long-term goal of developing an India-specific stroke risk prediction tool [12]. Given the scale and complexity of this study, a structured monitoring framework is essential to ensure systematic oversight, adherence to protocols, and uniformity across data collection sites. This paper describes the development and implementation of a multi-domain monitoring protocol designed explicitly for the NIMHANS–NH–SKAN Stroke study, detailing the processes, instruments, and indicators used to ensure high-quality data and robust governance.

## Methodology

### Study setting

The NIMHANS–NH–SKAN Stroke study comprises two components: a population-based cohort study and a quasi-experimental study. The cohort study consists of stroke-free and post-stroke groups. It investigates the role of risk factors in the occurrence of acute stroke and subsequent outcomes, including survival, disability, and quality of life. The quasi-experimental study component focuses on developing and evaluating a Comprehensive Acute Stroke CAre moDEl (CASCADE) designed to strengthen acute stroke management across multiple levels of the healthcare delivery system. The findings from both components are expected to inform stroke prevention, treatment pathways, health promotion strategies, and policy development in India. A detailed cohort methodology of the study has been published elsewhere [12]. A schematic representation of the processes involved in the development of the monitoring framework is provided in Fig 1.

**Fig 1:**
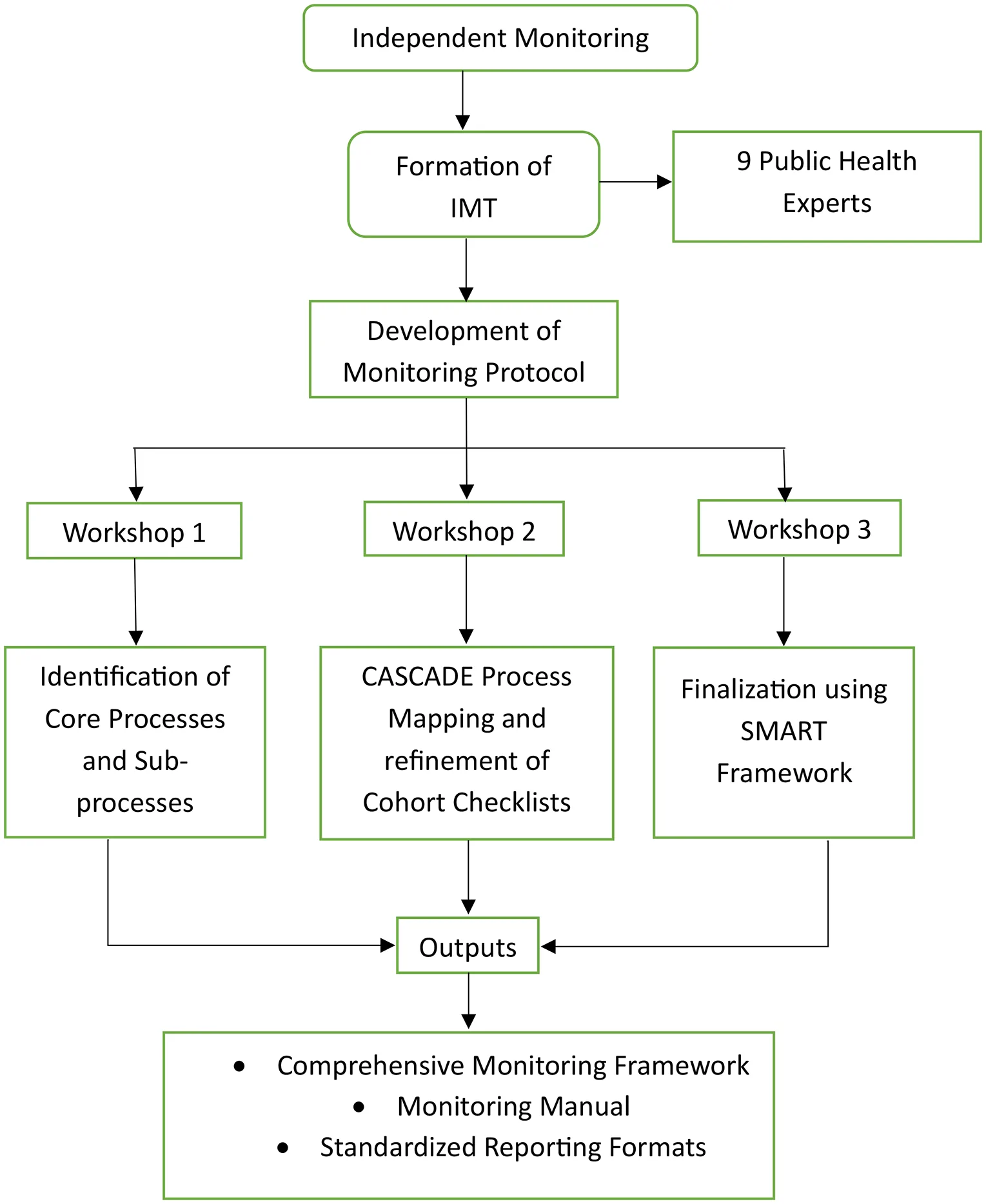
Schematic representation of monitoring process formation.

### Composition of the IMT

Formation of the IMT was guided by well-defined selection criteria aimed at ensuring technical expertise in terms of academic qualification, domain knowledge, familiarity with cohort study designs, longitudinal data collection, methodological skills, experience with data quality assurance, indicator development, or M&E (monitoring & evaluation) frameworks, and knowledge of research ethics. Further, operational efficiency, including field experience and supervisory experience, was considered desirable. A preliminary list of public health experts was generated and screened by the Principal Investigator and Co-Investigators according to the predefined eligibility criteria above. Nine experts were selected as IMT members, comprising seven men and two women. The IMT is responsible for providing independent oversight of study processes, field implementation, and adherence to the approved protocol.

### Development of the monitoring protocol

Given the scale and complexity of the NIMHANS–NH–SKAN project, an exclusive monitoring protocol was developed to guide systematic assessment of implementation fidelity, data quality, and ethical conduct. While both components were intended to be monitored under a single overarching monitoring framework developed for the project, separate monitoring tools were planned for each component. A comprehensive end-to-end monitoring framework was developed through three structured workshops held during the year 2023–2024, with a schedule for each workshop (S1). During the participatory development process, monitors and stakeholders identified, reviewed, and refined prioritized checklist items and indicators independently for the cohort and CASCADE components based on field-specific activities, implementation processes, protocol requirements, and data quality considerations relevant to each study design. It involved IMT members, project investigators, and the implementation team.

### Workshop 1: Identification of cohort processes and sub-processes

The first workshop focused on mapping core processes and sub-processes within the cohort component. A detailed overview of the project was presented. The Logical Framework Approach (LFA) guided the identification of monitoring processes by first mapping the expected outputs required for the cohort study. This was followed by the delineation of the implementation activities necessary to achieve these outputs. These implementation activities—recruitment, community engagement, case ascertainment, follow-up, data collection, sample handling, and data management—were treated as core processes for monitoring. Selection of activities was guided by the study protocol, with each activity included based on its direct contribution to a defined output of the cohort study. The sequence of activities reflected the study’s chronological workflow, from participant recruitment to data management, ensuring that monitoring processes aligned with the study’s operational structure. Further, IMT members were divided into four task groups, and each group decomposed the assigned process into sequential or component steps needed to execute it, yielding checklist items**.** This exercise generated a preliminary list of checklists and monitoring indicators. The sub-processes developed by each task group were compiled into a consolidated draft. An expert cross-review was subsequently undertaken, in which members of other task groups reviewed each task group's checklist. Reviewers suggested additional items, modifications, or removal of redundant items. Discrepancies or differences of opinion regarding the inclusion, wording, or classification of sub-processes were resolved through a plenary session by all members until agreement was reached.

### Workshop 2: Identification of CASCADE processes and application of the Vital Essential Desirable (VED) framework

Processes and sub-processes for the CASCADE component were identified using an approach similar to that described for the cohort component in Workshop 1: task-group-based decomposition of core processes by designated monitors, followed by consolidation, plenary discussion and consensus-based resolution of discrepancies. Every checklist item and indicator for cohort and CASCADE were revisited to ensure relevance, adequacy, and alignment with monitoring goals.

A Vital–Essential–Desirable (VED) strategy was applied for systematic prioritization:

- Vital: indispensable for ensuring participant safety, ethical compliance, regulatory adherence, and data integrity, where non-compliance could compromise the study

Essential: necessary for efficient implementation and quality assurance, but not immediately compromising the study.

Desirable: useful for strengthening processes, but not mandatory. Items not meeting the definition of “Vital” were excluded from the final list, thereby making monitoring efforts more efficient. The vital items were reviewed and, where required, refined for operational feasibility and endorsed through an open-ended discussion among all nine IMT members, with disagreements resolved iteratively until consensus was reached.

### Workshop 3: Finalization using the SMART framework

In the third workshop, all draft checklists and indicators were finalized using the SMART criteria (Specific, Measurable, Achievable, Relevant, Time-bound). The output of this workshop included:

1. One comprehensive monitoring framework consisting of a checklist along with items and indicators. For each indicator, the numerator, denominator and operational definitions were specified. All checklists were digitized using Google Forms. Skip patterns and automated calculations were embedded to compute indicators directly from entered field data. Separate forms were developed for each monitoring checklist, with dedicated formats for in-person observations and record reviews. Further, 2. A detailed monitoring manual: presenting the framework in an expanded, operational format—detailing how each item is to be assessed, verified, and documented during field visits; a structured monitoring schedule for field visits- specifying the frequency of visits, duration of field assessments, and the required number of IMT members for each activity, providing operational guidance for how the framework was to be applied in the field was drafted. 3. A standardized reporting format: structuring the findings from monitoring assessments into a consistent template for recording and communicating monitoring results across visits**.** This ensures uniform documentation across all monitoring visits and study components.

### Piloting and refinement of monitoring framework

The monitoring framework was applied during the study’s piloting phase, allowing it to be tested under real field conditions before full-scale implementation. During this phase, four IMT monitors conducted monitoring visits in accordance with the draft framework, checklist, and reporting format. The pilot aimed to assess the feasibility, usability, practicality, and completeness of the monitoring framework, including whether the checklist adequately covered all relevant aspects of the monitored processes. Based on issues identified during these visits—including [e.g., ambiguity in item wording, feasibility constraints, or gaps in indicator definitions]—modifications were made to the checklist and indicators. These modifications were subsequently reviewed and finalized by the full IMT, resulting in the finalized monitoring framework.

### Implementation of monitoring

The finalized monitoring framework, manual, and reporting format will be operationalized according to the schedule and procedures defined during framework development. Monitors will conduct site visits quarterly in accordance with the specified domains of assessment, verification methods, and minimum observation requirements outlined in the monitoring manual. During each visit, monitors will document observations, deviations, and good practices using the standardized reporting format and will provide recommendations where applicable. Findings from each visit will be shared with structured feedback mechanisms. Additionally, corrective actions are reviewed in subsequent monitoring cycles to ensure accountability.

## Results

Three monitoring workshops were conducted with participation from all nine members of the IMT. During the initial group activities, participants identified 112 checklist items and 58 indicators for the cohort component, 57 checklist items and 31 indicators for the CASCADE component. Following refinement, these were reduced to 56 checklist items and 34 indicators for the cohort component, and 31 checklist items and 15 indicators for the CASCADE model.

The application of the Vital–Essential–Desirable (VED) prioritization strategy further streamlined the tools to 59 checklist items and 28 indicators for the cohort component, and 26 checklist items with 12 indicators for the CASCADE component. After applying the SMART framework, the final monitoring checklist comprised 74 items and 13 indicators for the cohort study, 15 checklist items with three indicators for the CASCADE model. Overall, the finalized tools covered ten core processes (Table 1) and offered a comprehensive approach for monitoring implementation processes. To assess these processes, the target, numerator and denominator were specified for each key performance indicator (Table 2). All the targets were set based on project requirements.

**Table 1:** Summary of process checklists and indicators.

Sl No	Components	Process checklists	Purpose	Number of items	Number of indicators
1.	**Cohort**	Pilot study	To establish the feasibility and usability of study tools and procedures, facilitate early identification of potential challenges, and allow refinement before implementation	5	0
2.		Training	To ensure high-quality, standardized data collection, efficient operations, improved participant compliance and retention, and enhanced credibility	11	2
3.		Community mobilization	To enhance the reach and cooperation of the project among community members, fostering trust, facilitating recruitment, and supporting long-term retention of participants	10	4
4.		Data collection	To safeguard data quality, minimize protocol deviations, and maintain participant trust, ensuring the scientific integrity of the study and the rights of participants.	21	3
5.		Sample collection	To ensure protocol adherence, participant safety, and sample integrity	17	3
6.		Data management	To check data security procedures and confidentiality as per protocol	9	1
7.		Records and documentation	To ensure transparency and accountability	1	0
**TOTAL**				**74**	**13**
8.	**CASCADE**	Hospital preparedness	To enhance accuracy and credibility	6	1
9.		CASCADE model	To ensure protocol adherence	6	0
10.		Treatment and diagnostic algorithm	To monitor the feasibility of the CASCADE model in selected hospitals	3	2
**TOTAL**				**15**	**3**

**Table 2:** Description of operational processes and corresponding key performance indicators.

SL NO	Process	Key Performance Indicator	Target	Numerator	Denominator
1.	Training	Proportion of field staff who have completed training.	100%	Number of field staff trained	Number of field staff recruited
2.		Proportion of trainings where training reports are available	100%	Number of training reports available	Total number of trainings conducted
3.	Community mobilization	Proportion of villages or wards with at least one sensitization program being conducted	100%	Number of sensitization programs conducted in the village or wards	Total number of villages or wards where data collection has started or completed
4.		Proportion of IEC materials being displayed at prominent sites in the village/ward (where data collection has started)	100%	Number of villages where IEC materials were displayed in prominent sites	Total number of villages visited
5.		Proportion of randomly enquired community members who were aware of the program	80%	Number of randomly enquired community members who were aware of the program	Number of community members who were enquired about the awareness of the program
6.		Proportion of acute stroke events reported by the community members	NA	Number of acute stroke events reported by the community members	Total Number of acute stroke events reported
7.	Data collection	Proportion of functional electronic equipment	100%	Number of functional electronic equipment	Total number of electronic logistics
8.		Proportion of completed informed consent forms	100%	Number of completed informed consent forms	Total number of informed consent forms checked
9.		Proportion of data collectors adherent to SOP	100%	Number of data collectors adherent to SOP	Total number of data collectors observed
10.	Sample collection	Proportion of samples collected since the last IMT	80%	Number of participants who provided samples since the previous IMT	Total number of participants surveyed since the last IMT
11.		Proportion of samples drawn as per SOP	100%	Number of samples drawn as per SOP	Number of blood samples drawn observed
12.		Proportion of rejected samples	20%	Number of rejected samples	Total number of samples collected
13.	Data management	Proportion of queries resolved or addressed since the last IMT	100%	Number of queries resolved by the data team since the last IMT	Total number of queries received since the previous IMT visit
14.	Hospital preparedness	Proportion of the hospitals of each type where preparedness assessment was conducted	100%	Number of hospitals of each type where gap analysis was conducted	Total number of hospitals with each type
15.	Treatment and diagnostic algorithms	Proportion of hospitals with standard diagnostic and treatment algorithms implemented	NA	Number of hospitals with standard diagnostic and treatment algorithms implemented	Total number of hospitals with standard diagnostic and treatment algorithms
16.		Proportion of doctors who completed the training	NA	Number of doctors who completed the training	Total number of doctors recruited

*NA: Not Applicable (outside the scope of project management).

### Processes and sources of verification

Monitoring activities spanned multiple processes across both components of the study, with verification conducted using predefined checklists. It included a pilot study, training, community mobilization, data collection, sample collection, data management, records and documentation, hospital preparedness, the CASCADE model and treatment and diagnostics. All of these focused on ensuring strict adherence to protocol and procedures, and uniformity of processes.

Monitoring of Piloting aimed to review approved study tools. Monitoring of the training process focused on the availability and use of standardized training manuals, appropriateness of training methods, and adherence to scheduled training plans. Monitoring of community mobilization included key person sensitization, community sensitization programs, the functionality of the project helpline, social media engagement, and the display of IEC materials. Monitoring of data collection included verification of the availability of project IDs, permission letters, consent forms, medical and electronic equipment, stationery, and adherence to standardized consent and interviewing procedures. Completeness of consent forms and compliance with the protocol were assessed both in the field and through reviews of stored documents. Monitoring of sample collection covered adherence to biomedical waste management protocols, sample collection procedures, cold-chain maintenance, labelling accuracy, sample transportation, and storage. IMT data management and documentation assessments focused on database password protection, non-disclosure agreements (NDAs), regularity of data backups, and the volume and resolution of data queries. The availability of data management SOPs was also reviewed to ensure standardized and secure handling of study data. Maintenance of critical documents, including ethics approvals, Memoranda of Understanding (MoUs), consent forms, SOPs, and training manuals, was verified to ensure systematic and compliant documentation. Monitoring for the CASCADE component included reviewing hospital preparedness tools, standardized assessment reports, developing the CASCADE model, and verifying the completeness of diagnostic and treatment algorithms.

A monitoring manual specifying data sources and verification guidelines was drafted to ensure uniformity across monitors. Monitoring was decided to be conducted across community settings to monitor community mobilization, data and sample collection activities. Monitoring activities also included record reviews, documentation checks, data management and related activities at the project headquarters and field offices. It further included monitoring of laboratory-related procedures, including sample management, analysis, processing, storage, and reporting.

Verification of checklist items was decided to happen through multiple methods, including in-person observation, record review, interviews with community members, and visual observation. Some checklist items included multiple verification methods to facilitate triangulation. For example, completeness of consent forms was assessed by observing FDCs in the field and by randomly reviewing stored consent forms at the office. A detailed set of criteria was developed for each checklist item to address potential ambiguity and differences in interpretation across monitors. Minimum numbers were set for in-person observations (e.g., five interviews, two consent procedures, one sample collection, five IEC displays) to ensure comparability across sites and monitoring cycles. A remarks section was included in each checklist for monitors to record additional observations, emerging issues, or contextual insights.

### Monitoring schedule

Monitoring visits were planned quarterly, lasting 2–3 days and ensuring a minimum of four IMT members. The first monitoring visit was scheduled after 15–20 days of the commencement of data collection to enable early feedback and corrective actions.

### Reporting format

An indicator-based reporting format was adopted, enabling the systematic summarization of findings using predefined indicators. Progress was presented through dashboards that displayed current performance, targets, and trends across cycles, enabling rapid visualization of implementation processes. Each reporting format includes domain-wise analyses, deviations, actions taken to correct processes, good practices, bottlenecks and recommendations.

## Discussion

This article delineates the processes employed to develop and implement a structured independent monitoring framework for the NIMHANS-NH-SKAN stroke project. Additionally, the article discusses the importance of systematic monitoring in large-scale longitudinal research.

Historically, monitoring approaches in the Indian context were limited to domain-specific supervision. This underscored the need for a comprehensive, integrated monitoring framework. The results reaffirm that large-scale cohort studies require a multidimensional, iterative, and independent monitoring system rather than fragmented, component-specific approaches. Unlike short-term or single-site studies, longitudinal cohorts involve complex workflows spanning community engagement, recruitment, interview evaluation, biological sample management, and data management. Without structured monitoring, these interconnected systems are vulnerable to inconsistent implementation, protocol deviations, and compromised data integrity [18]. The development of an IMT protocol, therefore, addresses a critical need by providing a unified framework with independent oversight across diverse operational domains.

Globally, Data Monitoring Committees (DMCs) and Data and Safety Monitoring Boards (DSMBs) are considered the gold standard for oversight in randomized trials and biobank research [19,20]. However, their mandate is generally limited to interim safety and efficacy review, and their application in large-scale cohort studies and public health surveillance, particularly in low- and middle-income settings, has been limited. The IMT model described in this study addresses a distinct need within this context: rather than replicating a DSMB/DMC structure, it was independently designed to monitor the fidelity and quality of study implementation processes and the field complexity characteristic of the Indian public health environment. This work demonstrates that the IMT model can be successfully adapted in the above-stated circumstances. The IMT functions independently of the study investigators while maintaining constructive engagement with them, consistent with broader principles of independent research oversight that balance autonomy with collaborative feedback [21].

Unlike many existing monitoring systems for large-scale cohort studies, which are often developed informally or adapted from generic clinical trial checklists, the present framework was systematically derived through a structured process. This sequential, participatory approach—tested through real-world application during the survey piloting phase—ensured that the final framework was both methodologically grounded and field-tested before full-scale implementation, distinguishing it from ad hoc or retrospectively developed monitoring tools commonly used in similar studies.

While the specific checklist items and indicators were tailored to the components of this study, the underlying development process is readily generalizable to other large-scale cohort studies. This structured approach may be particularly valuable for multi-site or large-scale studies, where standardized, prioritized monitoring is essential to maintaining data quality across diverse field teams and locations. The workshop-based, iterative approach used to develop the monitoring framework underscores the value of participatory design in enhancing ownership, transparency, and contextual relevance. Involving investigators, IMT members, and field teams in the co-development of checklists, indicators, and reporting templates ensured both methodological rigor and operational feasibility. The structured use of the VED (Vital–Essential–Desirable) and SMART (Specific, Measurable, Achievable, Relevant, Time-bound) criteria enabled the systematic refinement of over 100 initial items into a concise, actionable set of indicators. Such consensus-driven approaches are rarely documented in Indian public health literature and represent a significant methodological advance [4,13]. While both components within this study, namely the cohort component and the CASCADE component, are monitored under a single overarching monitoring framework developed for the project, separate monitoring tools are created for each component. Thus, the framework provides a common structure for monitoring, whereas the actual checklist items and indicators are tailored to the unique monitoring needs of each study component. This approach ensures consistency in monitoring principles while maintaining methodological appropriateness for the distinct objectives and operational processes of the cohort and quasi-experimental components.

Digitalization of checklists represents another key strength of the framework. Digitalizing checklist forms supports real-time data capture, reduces transcription errors, and eases indicator computation. These features align with global best practices for adaptive monitoring [22].

The multi-site, multi-level monitoring further enhances the framework's robustness. IMT assessments included activities related to community mobilization, sample collection, data management and documentation. The use of multiple data sources, such as in-person observations, record reviews, interviews, and virtual assessments, reduced subjectivity by enabling triangulation. Further, minimum observation and explicit verification criteria improved consistency across monitors. It addresses the common challenge encountered in decentralized monitoring systems. It also aligns with the principles of RE-AIM and results-based management frameworks, which ensured validation across multiple sources [23].

In the broader Indian landscape, monitoring mechanisms within national programs such as NFHS, NTEP, and NACP have successfully strengthened data quality through structured supervision and verification [10,11,24]. However, these mechanisms remain program-driven and are not independently governed. They focus primarily on field-level fidelity rather than broader ethical, scientific, and methodological oversight. The IMT model presented here expands this paradigm by integrating ethical compliance, participant safety, sample handling, data security, and process fidelity within a single framework. This provides a scalable model for future large-scale research involving human participants.

The NIMHANS–NH–SKAN experience also demonstrates the feasibility of incorporating independent monitoring into institutional research systems. The composition of the IMT faculty, with expertise in epidemiology and community medicine, ensured methodological rigor while ensuring contextual sensitivity. Quarterly monitoring cycles, early feedback visits, and structured reporting, as envisaged, reinforce real-time course correction, reflecting the characteristics of “learning health systems” in which evidence continuously informs practice [25]. The framework was designed to be concise and specific yet comprehensive, balancing operational efficiency. Despite this, certain limitations are noteworthy. The inter-observer variability cannot be eliminated for items requiring qualitative assessments. Inter-rater reliability assessments can be incorporated into future frameworks. Furthermore, digital tools improve efficiency. However, connectivity issues in study sites may hamper real-time data uploads. Establishing an IMT involves additional financial commitments that may not be feasible for all research projects, particularly those operating under constrained budgets.

### Implications and future directions

The monitoring framework is integrated into the project protocol, including monitoring implementation resources or costs rather than being limited to the initial development and piloting phase. Monitoring activities are scheduled every three months throughout the study period. For sustainability, the project will retain ownership of the monitoring framework, while designated IMT members with relevant expertise will continue periodic monitoring. This monitoring framework represents a significant advance in operationalizing structured, independent oversight in Indian public health research. Its development process, built on participatory design, iterative refinement, and digital integration, offers a replicable and adaptable model for other large-scale studies. The IMT strengthens accountability and transparency while ensuring adherence to ethical and scientific standards.

However, the effectiveness of this framework in improving data quality, protocol adherence, and overall study rigor has not yet been formally evaluated. Future research should assess its impact on implementation outcomes. Given the resource requirements associated with deputing an Independent Monitoring Team, future studies should also evaluate the cost-effectiveness of this model relative to conventional or ad hoc monitoring approaches. Such evaluations would help establish the replicability, scalability and generalizability of this framework for adoption in other large-scale cohort studies.

## Conclusion

The structured monitoring framework developed for the NIMHANS–NH–SKAN Stroke Project addresses a critical methodological need in India’s public health research ecosystem. It demonstrates that the principles of independent, multi-domain monitoring—traditionally associated with clinical trials—can be effectively applied to population-based cohort studies. The participatory and risk-based approach described here provides a blueprint for future research to enhance implementation fidelity, ethical conduct, and data integrity. Institutionalizing such frameworks within research governance policies could substantially elevate the standards of public health research in India.

## Supporting information

S1 Table Schedule of the IMT workshops. (DOCX)

S2 Table Monitoring framework of NIMHANS-NH-SKAN Stroke Project. (DOCX)
