# Supplementary material for: Operationalizing independent oversight in large-scale public health cohorts: Monitoring protocol for the NIMHANS–NH–SKAN Stroke project
Source: PLoS One. 2026 Nov 4;21(11):e0361702. doi: 10.1371/journal.pone.0361702 (PMC13734449; doi:10.1371/journal.pone.0361702)
Supplement: Supplementary file 1 [file pone.0361702.s001.docx]

**S1 Table: Schedule of the IMT workshops**

| **1^st^ IMT workshop** | | |
| --- | --- | --- |
| **DAY 1 (27/03/2023)** | | **Resource Person/ Facilitation** |
| 10.00 am to 10.15 am | Registration |  |
| 10.15 am to 10.45 am | Introduction and overview of the NIMHANS- NH-SKAN project – Cohort Study | Project investigator |
| 10.45 am to 11.15 am | Introduction to an experimental study | Project investigator |
| 11.15 am to 11.30 am | Tea-break |  |
| 11.30 am to 12.00 pm | Need for an Independent monitoring team and terms of reference | Project investigator |
| 12.00 pm to 12.30 pm | Identification of monitoring processes | IMT + implementation team |
| 12.30 pm to 1.20 pm | Finalisation of monitoring processes. | IMT + implementation team |
| 1.20 pm to 2.00 pm | LUNCH |  |
| 2.00 pm to 4.30 pm | Group activity: Identification and Development of various monitoring tools and formats – for allocated processes | IMT + implementation team |
| **DAY 2 (28/03/2023)** | | **Resource Person/ Facilitation** |
| 9.45 am to 10.45 pm | Presentation of various monitoring tools and formats. Refinement and monitoring of tools and formats. | IMT + implementation team |
| 10.45 am to 11.00 am | Coffee break |  |
| 11.00 am to 1.20 pm | Presentation of remaining monitoring tools and formats. Refinement and monitoring of tools and formats. | IMT + implementation team |
| 1.20 pm to 2.00 pm | LUNCH |  |
| 2.00 pm to 3.30 pm | Presentation of remaining monitoring tools and formats. Refinement and monitoring of tools and formats. | IMT + implementation team |
| 3.30 pm to 3.45 pm | Group photo | ALL |
| 3.45 pm to 4.30 pm | Group Activity: Identification of Monitoring Indicators. | IMT + implementation team |
| 4:30 pm to 5.45 pm | Presentation and finalisation of monitoring indicators. | IMT + implementation team |
| **DAY 3 (29/03/2023)** | | **Resource Person/ Facilitation** |
| 10.00 am to 11.00 am | Presentation and finalisation of the remaining monitoring indicators. | IMT + implementation team |
| 11.00 am to 11.30 pm | Developing the outline for the IMT manual | IMT + implementation team |
| 11.30 pm to 12.30 pm | Writing the first draft of the IMT manual | IMT + implementation team |
| 12.30 pm-1.00 pm | Sharing of tentative dates for monitoring for the first year by the IMT members and identifying the nodal person of the independent monitoring team | IMT + implementation team |
| 1.00 pm-1.20 pm | Feedback session | ALL |
| 1.20 pm onwards | Lunch and farewell | ALL |
| **2^nd^ IMT workshop** | | |
| **DAY 1 (30/08/2023)** | | **Resource Person/ Facilitation** |
| 9 am to 9.15 am | Inauguration of the programme | Project investigator |
| 9.15 am to 9.45 am | Overview of the 1^st^ IMT workshop and CASCADE component overview | Project investigator |
| 9.45 am to 10.30 am | Identification of monitoring processes | IMT +  implementation team |
| 10.30 am to 10.45 am | Tea/Coffee break |  |
| 10.45 am to 11.30 am | Finalisation of monitoring processes. | IMT +  implementation team |
| 11.30 am to 1.00 pm | Group activity: Identification and Development of various monitoring tools and formats – for the allocated processes | IMT +  implementation team |
| 1.00 pm to 2.00 pm | LUNCH |  |
| 2.00 pm to 3.30 pm | Presentation of various monitoring tools and formats. Refinement and monitoring of tools and formats. | IMT +  implementation team |
| 3.30 pm to 3.45 pm | Tea/Coffee break |  |
| 3.45 pm to 4.30 pm | Group Activity: Identification of Monitoring Indicators. | IMT +  implementation team |
| **DAY 2 (31/08/2023)** | | **Resource Person/ Facilitation** |
| 9.15 am to 9.30 am | Group photo | ALL |
| 9.30 am to 10.30 pm | Presentation and finalisation of the remaining monitoring indicators. | IMT +  implementation team |
| 10.30 am to 10.45 am | Tea/Coffee break |  |
| 10.45 am to 11.30 pm | VED strategy application to develop monitoring tool formats | IMT +  implementation team |
| 11.30 pm to 1.00 pm | VED strategy application to develop monitoring tool formats | IMT +  implementation team |
| 1.00 pm to 2.00 pm | LUNCH |  |
| 2.00 pm to 3.30 pm | VED strategy application to develop monitoring tool formats | IMT +  implementation team |
| 3.30 pm to 3.45 pm | Tea/Coffee break |  |
| 3.45 pm to 4.30 pm | VED strategy application to develop monitoring tool formats | IMT +  implementation team |
| **DAY 3 (01/09/2023)** | | **Resource Person/ Facilitation** |
| 10.00 am to 11.00 am | Building on the outline of the monitoring manual development | IMT +  implementation team |
| 11.00 am to 11.15 am | Tea/Coffee break |  |
| 11.15 am to 12.45 pm | Presentation of finalised monitoring manual | IMT +  implementation team |
| 12.45 pm to 1.00 pm | Sharing of tentative dates for the subsequent monitoring of the next IMT | IMT +  implementation team |
| 1.00 pm onwards | Lunch and farewell | ALL |
| **3^rd^ IMT workshop** | | |
| **DAY 1 (27/06/2024)** | | **Resource Person/ Facilitation** |
| 9.30 am to 10.00 am | Brief about the current progress of the project: Introduction | Project investigator |
| 10.00 am to 10.30 am | SMART criteria application to the developed monitoring formats | IMT +  implementation team |
| 10.30 am to 10.45 am | Tea/Coffee break |  |
| 10.45 am to 1.00 pm | SMART criteria application to the developed monitoring formats | IMT +  implementation team |
| 1.00 pm to 2.00 pm | LUNCH |  |
| 2.00 pm to 3.00 pm | SMART criteria application to the developed monitoring formats | IMT +  implementation team |
| 3.00 pm to 3.15 pm | Tea/Coffee break |  |
| 3.15 pm to 4.30 pm | SMART criteria application to the developed monitoring formats | IMT +  implementation team |
| **DAY 2 (28/06/2024)** | | **Resource Person/ Facilitation** |
| 9.15 am to 10.30 am | Digitisation of developed monitoring formats | IMT +  implementation team |
| 10.30 am to 10.45 am | Tea/Coffee break |  |
| 10.45 am to 1.00 pm | Development of a standardized reporting format | IMT +  implementation team |
| 1.00 pm to 2.00 pm | LUNCH |  |
| 2.00 pm to 3.00 pm | Debriefing and group Photo | ALL |
