# Supplementary material for: Operationalizing independent oversight in large-scale public health cohorts: Monitoring protocol for the NIMHANS–NH–SKAN Stroke project
Source: PLoS One. 2026 Nov 4;21(11):e0361702. doi: 10.1371/journal.pone.0361702 (PMC13734449; doi:10.1371/journal.pone.0361702)
Supplement: Supplementary file 2 [file pone.0361702.s002.docx]

**Supplementary file 2: Monitoring framework of NIMHANS-NH-SKAN Stroke Project**

| **SL NO** | **Monitoring item** | **Indicator** | **Data source** | **Mode of Monitoring** | **Target** | **Corrective action** |
| --- | --- | --- | --- | --- | --- | --- |
|  | **Process /Activity:** Pilot study | | | | | |
|  | **Frequency:** One-time activity before the beginning of the actual study**.** **Research Officer** is the responsible person. | | | | | |
| 1 | Was the pilot study conducted as per protocol? | NA | Reports and Photographs | Record Review | Pilot study to be conducted, adhering to the approved protocol | Repeat the piloting |
| 2 | Were there any modifications in the study protocol after the pilot study? | NA | IEC modifications | Record Review | Modification to be made and ethical approval to be obtained | Ensure all procedural approvals. |
| 3 | Is the standard report of pilot study available? | NA | Piloting report | Record Review | Modifications, challenges and deviations to be documented | Ensure prompt documentation of all piloting. |
| 4 | Are all the study questionnaires piloted? | NA | Piloting reports | Record Review | All study tools to be piloted | Conduct piloting. |
| 5 | Are the study questionnaire reviewed and modified following the pilot study? | NA | Record Review | Record Review | All discrepancies to be documented and modified. Modified questions to be updated in the questionnaire | Update the questionnaire. |
|  | **Process /Activity:** Training of FDCs | | | | | |
|  | **Frequency:** Every visit if there are new FDCs recruited since the last IMT. **Research Officer** is the responsible person. | | | | | |
| 6 | Number of training programs conducted since the last IMT | NA | Training reports | Record review | Induction training for all FDCS | To refrain from field activities and provide training |
| 7 | Number of field staff who completed training since the last IMT | Proportion of field staff who have completed training (with min of 60% post-test scores). | Training reports | Record Review | All field staff to be compulsorily trained | To conduct training |
| 8 | Are the training manuals available in Kannada and English? | NA | Manual Review | Record Review | To enable easy access and reference to FDCs whenever needed | Translate and provide the training manuals in both Kannada and English to all FDCs |
| 9 | Are the field staff trained using these manuals? | NA | Lesson plans in training reports | Record review | To enable easy access and reference to FDCs whenever needed | Conduct training based on manuals |
| 10 | Number of field staff recruited since the last IMT | NA | Attendance register | Record Review | FDC positions are maintained at full strength at all times | Recruit FDCs |
| 11 | Compiled data of FDCs' pre- and post-test scores available in digital format? | NA | Training reports | Record Review | Selection of FDCs achieving ≥60% in the post-test | Conduct booster training to FDCs with post-test score <60% |
| 12 | Whether refresher training is provided as and when required periodically | NA | Training reports | Record Review | Have at least 1 refresher training program annually | To ensure refresher trainings are conducted. |
| 13 | Is training imparted as per the training schedule? | NA | Schedule in training reports | Record Review | All trainings to be imparted as per the schedule | Reschedule missed or delayed sessions and monitor implementation. |
| 14 | Are the teaching-learning methods adopted for training appropriate? | NA | Training reports | Record Review | To make training didactic | Modify training methods |
| 15 | Are the training reports with photographs available for verification? | NA | Training reports | Record review | Documentation | Ensure taking geo-tagged photographs  are taken in future trainings. |
| 16 | Number of training reports with photographs available for verification | Proportion of trainings where training reports are available | Record Review | Record Review | Documentation of all training programs | Prepare and maintain training reports with photographs for all completed training sessions |
|  | **Process /Activity:** Community mobilisation | | | | | |
|  | **Frequency:** Every visit if community mobilisation activities were conducted since the last IMT**. Community mobiliser** is the responsible person. | | | | | |
| 17 | IEC materials with relevant content displayed in local language at prominent places in study area | Proportion of IEC materials being displayed at prominent sites in the village or wards (where data collection has started) | Field observation | In-person verification | IEC materials to be displayed in all prominent places | Ensure IEC materials are displayed at least 3 prominent places |
| 18 | Use of any social media (Facebook/ Twitter/ Instagram/ WhatsApp/ YouTube) and/or mass media for sensitisation? | NA | Verification of social media and/or mass media | Digital platform review | Regular update | Regular social media engagement |
| 19 | Number of acute stroke events reported by the community members (includes ASHAs, ANMs, AWWs, family members, neighbours, village leaders) | Proportion of acute stroke events reported by the community members | Repository of case records | Record review | acute stroke events in the community identified and reported through continuous updates from community informants (ASHAs, ANMs, AWWs, family members, neighbours and village leaders). | Strengthen the active surveillance. Strengthen regular communication and follow-up with community informants to identify and report any missed stroke events promptly. |
| 20 | Total no. of acute stroke events reported by the community members | NA | Repository of case records | Record review | All cases to be reported by community members. | Strengthen the surveillance |
| 21 | Helpline number available and functional | NA | Working helpline mobile handset | In-person/online | Helpline number available and functional at all times. | Regularly test the helpline and promptly address any connectivity or operational issues. |
| 22 | No of community members who were interviewed about the awareness of the program | NA | Field observation | In-person | NA | NA |
| 23 | No. of randomly interviewed community members who were aware about the program | Proportion of randomly enquired community members aware of the program | Field observation | In-person | All community members should be aware of the survey | Adapt different ways of Sensitisation to reach the maximum population. Improve content quality of program delivery |
| 24 | Was there at least one Sensitization sessions for the following groups before data collection: i. key opinion leaders & community leaders ii. grass root health workers iii. district administrative authorities iv. community members | Proportion of villages or wards with at least one sensitization program being conducted for community members (key opinion leaders/grass root health workers/ community/ district administrative authorities) where data collection is started or completed | Record review | Record review | Reaching all key community persons | To ensure at least one sensitization program to be conducted |
| 25 | Total number of villages or wards where data collection started | NA | Record review | Record review | Number of villages to be covered as per plan | Revise the plan to meet the deadline. |
| 26 | Reports of Sensitization Programs conducted are available. If yes, specify the number | NA | Sensitization program register | Record review | Documentation of all sensitization programs | Prepare and maintain training reports with photographs for all completed sensitization programs. |
|  | **Process /Activity:** Data collection | | | | | |
|  | **Frequency:** Every visit if data collection activities were conducted since the last IMT**. Research officer** is the responsible person. | | | | | |
| 27 | No. of data collectors observed- household questionnaire and individual questionnaire | NA | Field observation | In-person | NA | NA |
| 28 | Number of participants surveyed since last IMT (Household and individual) | NA | Data management system | Record review | Pace of data collection to be maintained | Root cause analysis |
| 29 | Does the data collector have project ID? | NA | Field observation | In-person | All data collectors must have an ID card | Ensure ID cards are provided to all field staff |
| 30 | Availability of permission letter with data collector | NA | Field observation | In-person | All data collectors must have permission letters | Ensure necessary permission letters are with data collectors. |
| 31 | Availability of the following functional medical equipment with FCs i. BP apparatus ii. Weighing scale iii. Stadiometer | NA | Field observation | In-person | All the medical equipment must be functional | Mandatory regular calibration and checking of all medical equipment |
| 32 | Availability of functional electronic equipment iv. Digital tablet/mobile v. Charger vi. Power bank | Proportion of functional electronic equipment | Field observation | In-person | All the electronic equipment must be functional | Maintaining a checklist of equipment |
| 33 | Availability of stationary logistics vii. ink pad viii. pen | NA | Field observation | In-person | All stationery logistics to be functional | Maintaining a checklist of logistics |
| 34 | Availability of adequate number of unfilled Informed Consent forms | NA | Field observation | In-person | Carry the required number of unfilled Informed Consent Forms plus 30% additional forms | Micro-planning and checklist. |
| 35 | Availability of hard copy of questionnaires with the data collector | NA | Field observation | In-person | 10 hard copy questionnaires to be carried | Ensure unfilled hard copy of questionnaires in checklist |
| 36 | Did the data collector ensure privacy confidentiality while collecting data? | NA | Field observation | In-person | Privacy to be ensured during interview | Reinforce regarding interview ethics: privacy and confidentiality |
| 37 | Did the data collector completely explain the project in the local language? | NA | Field observation | In-person | Proper explanation of all the details of ICF | Training and handholding |
| 38 | Was the Interviewer adherent to SOP of data collection, throughout the interview? | NA | Field observation | In-person |  | Retraining and reinforcing |
| 39 | No. of data collectors adherent to SOP- household questionnaire and individual questionnaire | Proportion of data collectors adherent to SOP | Field observation | In-person | All data collectors must adhere to SOP | Retraining |
| 40 | Total number of data collectors collecting the data | NA | Attendance register | Record review | All data collector positions should be filled | Recruitment |
| 41 | No. of signed informed consent forms since last IMT. (Household and individual) | NA | Data management system | Record review | All data collected must have informed consent form | Retrieve the uploaded consent form and store it manually |
| 42 | Total no of informed consent forms checked since last IMT (with unique ID) | NA | Stored consent forms | Record review | All informed consent forms should be complete | Repeat consent or discard the data |
| 43 | Are the informed consent forms complete? Mention the numbers | Proportion of completed informed consent forms | Observation and record review | Record review | All informed consent forms to be completed | Re-training |
| 44 | Are signed informed consents stored digitally? | NA | Data management system | Record review | All informed consent forms to be stored digitally | Store digitally |
| 45 | Are signed informed consents stored manually? | NA | Field observation | Record review | All informed consent forms to be stored digitally | Store manually |
| 46 | Is first aid kit available in field? | NA | Field observation | In person | First-aid kit available with the field team, with all medicines and supplies within their expiry date. | Provide first-aid kit. |
| 47 | Number of interviews conducted using hard copies | NA | Field observation | In person | Minimise the use of data collection using hard copies of questionnaire. | Ensure any data collected on hard copies is promptly and completely entered into the digital data collection system, with the hard-copy record retained as required. |
|  | **Process /Activity:** Sample collection | | | | | |
|  | **Frequency:** Every visit if sample collection activities were conducted since the last IMT**. Research officer** is the responsible person. | | | | | |
| 48 | No. of participants who consented to provide samples since last IMT | NA | Data management system | Record review | 100% of interviewed participants should provide informed consent for blood sample collection. | Identify the root cause for non-consent and strengthen skills to address. |
| 49 | No. of participants from whom blood samples were collected since last IMT: EDTA | Proportion of samples collected since last IMT | Data management system | Record review | Blood samples collected from 100% of participants who provide consent for sample collection. | Mop-up rounds/revisiting |
| 50 | No. of participants from whom blood samples were collected since last IMT: SST | NA | Data management system | Record review | Blood samples collected from 100% of participants who provide consent for sample collection. | Mop-up rounds/revisiting |
| 51 | No of phlebotomists observed | NA | Field observation | In person | NA | NA |
| 52 | No of samples drawn observed | NA | Field observation | In person | Zero errors in observed blood sample collection procedures. | Supportive supervision and hand holding. |
| 53 | Are the phlebotomists carrying sufficient consumables to collect blood samples for the day for collecting blood sample? | NA | Field observation | In person | Carry the required number of blood sample collection consumables plus 30% additional consumables as a buffer | Micro-planning and checklist. |
| 54 | Is the lab technician adhering to SOPs while drawing blood sample? | Proportion of samples drawn as per SOP | Field observation | In person | Zero deviations from SOP | Reinforcing the SOPs |
| 55 | Are the blood samples appropriately labelled? | NA | Field observation | In person | 100% of collected blood samples to be appropriately labelled | Label or Re-label accordingly |
| 56 | Are the blood samples transported as per SOP? | NA | Record review | In person | 100% adherence to the SOP | Reinforcing the SOPs |
| 57 | Is Bio-Medical Waste management procedures done as per SOP? | NA | Field observation | In person | 100% adherence to the SOP | Reinforcing the SOPs |
| 58 | Are the samples stored as per SOP for cold chain management? | NA | Field observation | In person | 100% adherence to the SOP | Reinforcing the SOPs |
| 59 | Number of blood samples drawn as per SOP | NA | Field observation | In person | Zero deviations from SOP | Reinforce |
| 60 | No. of needle stick injuries reported | NA | Needle stick injuries register | Record review | All needle stick injuries to be reported. | Sensitize reporting mandatory |
| 61 | Number of blood samples rejected from laboratory since last IMT: EDTA | Proportion of rejected samples | Data management system | Record review | Zero rejection of blood samples | Training and ensuring resampling |
| 62 | Number of blood samples rejected from laboratory since last IMT: SST | NA | Data management system | Record review | Zero rejection of blood samples | Training and ensuring resampling |
| 63 | Are the investigation reports shared with study participants regularly? | NA | Data management system | Record review | All reports to be shared regularly. | Build a mechanism for internal monitoring regularly |
| 64 | Total number of reports shared with study participants since last IMT | NA | Data management system | Record review | All reports of blood samples collected to be shared | Build a mechanism for internal monitoring regularly |
|  | **Process /Activity:** Data management**.** | | | | | |
|  | **Frequency:** Every visit after data collection has started**. Data manager** is the responsible person. | | | | | |
| 65 | Is the Non-Disclosure Agreement (NDA)signed by every staff working in the project |  | Records and reports | Record review | Every staff member should have signed the NDA | Ensure NDAs are obtained from staff with missing signatures |
| 66 | Are SOPs available with the data management team w.r.t data backup, security, storage? | NA | SOPs | Record review | All SOPs should be available and accessible | Identify missing or outdated SOPs and update them. |
| 67 | Is data entry manual available for digital data collection? | NA | Data entry manual | Record review | Data entry manual to be available | Prepare a data entry manual. |
| 68 | Whether data back-up has been taken daily? | NA | Hard disc | Record review | 100% of data backups are completed daily | Identify the reason for any missed backup and ensure the backup is completed immediately |
| 69 | Are SOPs available to address the technical glitch? | NA | SOPs | Record review | All SOPs should be available and accessible | Identify missing or outdated SOPs and update them. |
| 70 | Is the database password protected? | NA | Hard disc | Record review | Database is strong password protected with access restricted to authorised personnel only | Ensure password protection and restrict open access |
| 71 | Number of queries raised by data management team | NA | Excell document | Record review | NA | NA |
| 72 | Number of queries resolved by data management team | Proportion of queries resolved/addressed since last IMT visit | Excell document | Record review | All queries raised to be resolved by data management team | Review the queries weekly and resolve |
| 73 | Whether the data accuracy is checked by field coordinator | NA | Data quality check register | Record review | 10% of data collected by the data collector to be quality checked by field coordinator | Ensure the Field Coordinator completes the required 10% quality checks. |
|  | **Process /Activity:** Record and documentation | | | | | |
|  | **Frequency:** Every visit**. Administrative officer** is the responsible person. | | | | | |
| 74 | Are documents maintained as per the protocol? (Critical documents per component will be asked whether document is available) | NA | Records | Record review | Documents to be maintained as per protocol. | Maintain documents. |
|  | **Process /Activity:** Hospital preparedness | | | | | |
|  | **Frequency:** Every visit if hospital preparedness activities have been undertaken since last IMT**. Research officer** is the responsible person. | | | | | |
| 75 | Availability of tool for assessing hospital preparedness? | NA | Hospital preparedness tool | Record review | Availability of a complete questionnaire | Develop a tool |
| 76 | Whether tool has covered all components of hospital preparedness? | NA | Hospital preparedness tool | Record review | All the components are included in the tool | Include the missed components |
| 77 | Are the SOPs, algorithms/manuals developed for data collection? | NA | SOPs, algorithms/manuals | Record review | Availability of all SOPs, algorithms/manuals | Prepare non-available SOPs, algorithms/manual |
| 78 | Were the data collectors trained for hospital preparedness assessment? | NA | Training report | Record review | All data collectors should be trained | Training |
| 79 | Whether preparedness was assessed in all hospitals mentioned in the protocol? | Proportion of hospitals where preparedness assessment was conducted | Collected data record | Record review | Should be assessed at all study hospitals | To conduct the assessment |
| 80 | Whether any other hospitals apart from mentioned in the protocol were assessed as a part of project? | NA | Collected data record | Record review | NA | NA |
|  | **Process /Activity: CASCADE MODEL** | | | | | |
|  | **Frequency:** Every visit if CASCADE model development has been undertaken since last IMT**. Research officer** is the responsible person. | | | | | |
| 81 | Whether SOPs for screening are available? | NA | SOPs | Record review | Availability of SOPs | Prepare SOPs |
| 82 | Whether SOPs for triage system are available? | NA | SOPs | Record review | Availability of SOPs | Prepare SOPs |
| 83 | Whether case management/treatment/diagnostic algorithms are available? | NA | Algorithms | Record review | Availability of SOPs | Prepare SOPs |
| 84 | Are SOPs for referral available? | NA | SOPs | Record review | Availability of SOPs | Prepare SOPs |
| 85 | Whether healthcare personnel were trained in triage system? | NA | Training reports | Record review | All healthcare personnel should be trained | Provide training |
| 86 | Whether hospital personnel were trained on CASCADE model? | NA | Training reports | Record review | All healthcare personnel should be trained | Provide training |
|  | **Process /Activity: Treatment and diagnostic algorithms** | | | | | |
|  | **Frequency:** Every visit if CASCADE model development has been undertaken since last IMT**. Research officer** is the responsible person. | | | | | |
| 87 | Whether a team of experts were involved in developing standard diagnostics and treatment algorithms following a consultative process? | Proportion of hospitals with standard diagnostic and treatment algorithms implemented | Authors and contributor list of the model or meeting minutes of consultative workshop | Record review | Should be involved | Get it reviewed by experts |
| 88 | No. of doctors recruited in study hospitals | NA | Hospital attendance register | Record review | All doctors to be trained | Conduct training |
| 89 | No. of doctors trained in study hospitals | Proportion of doctors who completed training | Hospital training reports | Record review | All doctors to be trained | Conduct training |
